# High prevalence of epilepsy in onchocerciasis endemic regions in the Democratic Republic of the Congo

**DOI:** 10.1371/journal.pntd.0005732

**Published:** 2017-07-14

**Authors:** Bethany Levick, Anne Laudisoit, Floribert Tepage, Chellafe Ensoy-Musoro, Michel Mandro, Caroline Bonareri Osoro, Patrick Suykerbuyk, Jean Marie Kashama, Michel Komba, Alliance Tagoto, Dadi Falay, Michael Begon, Robert Colebunders

**Affiliations:** 1 Institute of Integrative Biology, School of Biological Sciences, University of Liverpool, Liverpool, United-Kingdom; 2 CIFOR, Jalan Cifor, Situ Gede, Sindang Barang, Bogor Bar, Jawa Barat, Indonesia; 3 Global Health Institute, University of Antwerp, Antwerp, Belgium; 4 National Onchocerciasis Control Program (PNLO), Ministry of Health, Buta, Democratic Republic of the Congo; 5 Interuniversity Institute for Biostatistics and statistical Bioinformatics, University of Hasselt, Hasselt, Belgium; 6 Ituri Provincial Health Division, Ministry of Health, Bunia, Democratic Republic of the Congo; 7 Nanyuki Teaching and Referral Hospital, Nanyuki, Kenya; 8 Neuropsychopathologic Centre of Mont Amba (CNPP), University of Kinshasa, Kinshasa, Democratic Republic of the Congo; 9 Biodiversity Monitoring Centre, Faculty of Sciences, University of Kisangani, Kisangani, Democratic Republic of the Congo; 10 National HIV program, Ministry of Health, Kisangani, Democratic Republic of the Congo; 11 Department of Pediatrics, University of Kisangani, Kisangani, Democratic Republic of the Congo; Instituto de Investigaciones Biomedicas, UNAM /Instituto de Neurologia y Neurocirugía, MEXICO

## Abstract

**Background:**

An increased prevalence of epilepsy has been reported in many onchocerciasis endemic areas. The objective of this study was to determine the prevalence of epilepsy in onchocerciasis endemic areas in the Democratic Republic of the Congo (DRC) and investigate whether a higher annual intake of Ivermectin was associated with a lower prevalence of epilepsy.

**Methodology/Principle findings:**

Between July 2014 and February 2016, house-to-house epilepsy prevalence surveys were carried out in areas with a high level of onchocerciasis endemicity: 3 localities in the Bas-Uele, 24 in the Tshopo and 21 in the Ituri province. Ivermectin uptake was recorded for every household member. This database allowed a matched case-control pair subset to be created that enabled putative risk factors for epilepsy to be tested using univariate logistic regression models. Risk factors relating to onchocerciasis were tested using a multivariate random effects model. To identify presence of clusters of epilepsy cases, the Kulldorff's scan statistic was used. Of 12, 408 people examined in the different health areas 407 (3.3%) were found to have a history of epilepsy. A high prevalence of epilepsy was observed in health areas in the 3 provinces: 6.8–8.5% in Bas-Uele, 0.8–7.4% in Tshopo and 3.6–6.2% in Ituri. Median age of epilepsy onset was 9 years, and the modal age 12 years. The case control analysis demonstrated that before the appearance of epilepsy, compared to the same life period in controls, persons with epilepsy were around two times less likely (OR: 0.52; 95%CI: (0.28, 0.98)) to have taken Ivermectin than controls. After the appearance of epilepsy, there was no difference of Ivermectin intake between cases and controls. Only in Ituri, a significant cluster (p-value = 0.0001) was identified located around the Draju sample site area.

**Conclusions:**

The prevalence of epilepsy in health areas in onchocerciasis endemic regions in the DRC was 2–10 times higher than in non-onchocerciasis endemic regions in Africa. Our data suggests that Ivermectin protects against epilepsy in an onchocerciasis endemic region. However, a prospective population based intervention study is needed to confirm this.

## Author summary

An increased prevalence of epilepsy has been reported in many onchocerciasis endemic areas. Between July 2014 and February 2016, house-to-house epilepsy prevalence surveys were carried out in the Bas-Uele, Tshopo and Ituri province of the Democratic Republic of the Congo, in areas with a high level of onchocerciasis endemicity. Of 12, 408 people examined in the different health areas 407 (3.3%) were found to have a history of epilepsy. A nested case control analysis demonstrated that before the appearance of epilepsy, compared to the same life period in controls, persons with epilepsy were around two times less likely to have taken Ivermectin than controls. Our data suggests that Ivermectin protects against epilepsy in an onchocerciasis endemic region. However, a prospective population based intervention study is needed to confirm this.

## Introduction

A high prevalence of epilepsy (>1%) has been reported in many onchocerciasis endemic regions. In Africa, a special form of epilepsy referred to as “nodding syndrome” has been reported solely in onchocerciasis endemic areas [1]. The Democratic Republic of the Congo (DRC) is the country with the largest population at risk for onchocerciasis, with approximately 40.3 million people exposed and 29.7 million currently treated [2,3]. In the DRC, despite 16 years of Community Directed Treatment with Ivermectin (CDTI), the therapeutic coverage of Ivermectin is not consistent spatially or from year to year, and some areas remain untreated (have received no CDTI at all). According to the WHO, the geographic coverage for the country in 2007 was 72.2% and the therapeutic coverage 43.5% and in 2015, 93.3% and 73.3% respectively [3,4]. The inconsistent therapeutic coverage combined with the high level of onchocerciasis endemicity, may thus have direct consequences for the epilepsy prevalence in endemic areas in the DRC. In 2014, we documented the prevalence of epilepsy to be 2.9% in the village of Dingila and 2.3% in Titule, both in the Bas-Uele province [5]. In Titule, epilepsy showed a marked spatial pattern with clustering of cases occurring within and between adjacent households. Individual risk of epilepsy was found to be associated with living close to the nearest fast flowing river where blackflies (Diptera: Simuliidae)–the vector of *Onchocerca volvulus* (*O*.*v*)–oviposit and breed [5]. A small case control study in Titule suggested that Ivermectin may protect against onchocerciasis associated epilepsy or OAE [6].

In this study, we investigated in door-to-door village surveys whether there was increased epilepsy prevalence in other onchocerciasis endemic foci of the DRC, in the provinces of Bas-Uele, Tshopo and Ituri (previously part of the “Oriental Province”). Moreover, in a nested case control study we examined whether there was evidence for annual intake of Ivermectin being able to provide significant protection against epilepsy.

## Materials and methods

Definition: A case of epilepsy was defined as a patient who reported at least 2 unprovoked seizures without fever or any acute illness [7].

### Estimation of the epilepsy prevalence

**Fig 1:**
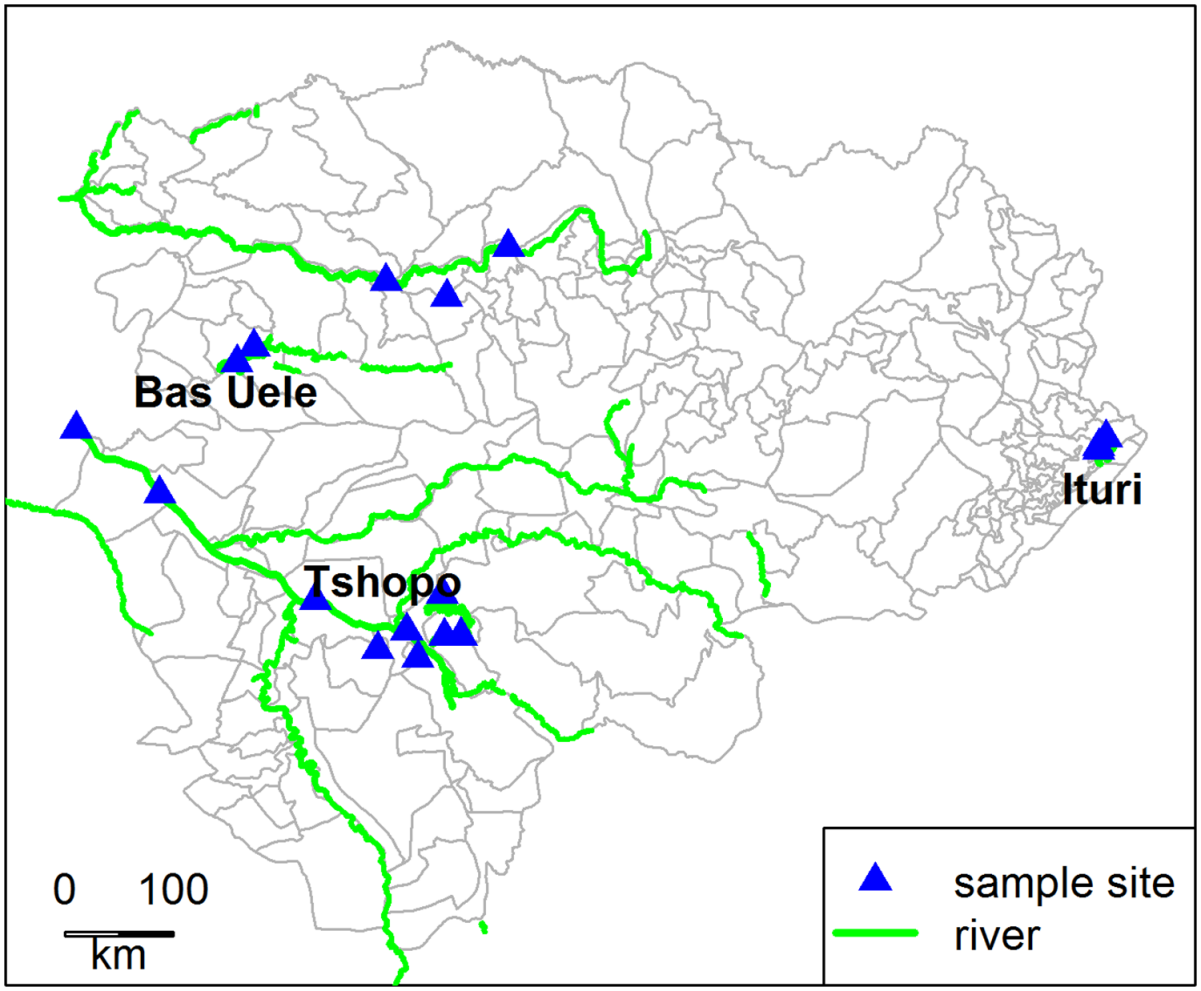
Location of the study sites in the former Oriental Province of the Democratic Republic of the Congo.

Between July 2014 and February 2016, house-to-house epilepsy prevalence surveys were carried out in 3 localities of Bas-Uele covering 164 households, 24 localities of Tshopo covering 1322 households and 19 localities of Ituri province covering 570 households (Fig 1).

The localities were selected on the basis of a historically high level of onchocerciasis endemicity based on Rapid Epidemiological Monitoring of Onchocerciasis (REMO) (1998 or 2003) data provided by the National Onchocerciasis control program (Programme National de Lutte contre l’Onchocercose, PNLO) [8]. In Ituri localities within one health area were chosen because they had never been included in the CDTI program and as such never received Ivermectin.

In the three provinces, houses were selected starting from the localities centres’, selecting every third household. If household members of the selected house were not at home, the next house was visited; all households were geo-located (handheld Garmin 62Cs GPS; ±4m accuracy). This method was implemented in all study sites except in the Tshopo province, in the Wanierukula health zone, where all houses on a 40km stretch of the national road linking Kisangani (Tshopo province) to the Ituri province were visited. The latter region is referred to as the “PK30-PK70” region (as it does not exist, administratively) and contains 1182 unique households across 2 health areas consisting of 14 localities. In every area, all household heads and parents of children present in the household were interviewed in their local language. For every consenting household, a one page questionnaire was completed (available as supporting information). Age and sex of every household member and their individual history of Ivermectin uptake each year between 2000–2015 were recorded. Screening for epilepsy was performed using the five questions questionnaire validated by Diagana et al [9]. The research team who visited the households consisted of one or two local health care workers or Ivermectin community distributers and a medical doctor. If a person with epilepsy was identified, family members were asked by a doctor to describe the type of seizures (or to show what happens during a seizure), to report on the precipitant circumstances, the duration of seizures, whether they were associated with uncontrollable tongue biting or passing of urine or stool, whether there were episodes of absence (sudden episodes of decreased consciousness of sudden onset and short duration) with or without nodding of the head. Questions were asked in the local language Boa, Lingala, Kiswahili or Lendu according to the site. The final diagnosis of epilepsy was made by a medical doctor.

For household members with epilepsy confirmed by the doctor, the age of the first epilepsy episode was noted. For those who developed epilepsy recently, the exact month of the first epilepsy attack was recorded. Onset of epilepsy was considered recent if a first seizure appeared in the 12 months before the survey date.

The prevalence of epilepsy in the populations surveyed was calculated across selected health areas of the three provinces (Ituri, Tshopo, and Bas-Uele). The data on the prevalence of skin onchocerciasis lesions (leopard skin) and therapeutic coverage were obtained through household interviews. The prevalence of people with onchocerciasis nodules (determining the level of onchocerciasis endemicity) were either obtained from the PNLO database or measured in the field using the standard REMO procedure [8]. The number of years of Ivermectin distribution in each health area was also obtained from the PLNO database. Ivermectin coverage per health area was calculated as the number of individuals in the health are who reported to have taken Ivermectin in 2014 over the number of individuals eligible to receive Ivermectin in that year. As eligibility criterium for Ivermectin treatment we only used age > 5 years old; we did not took into account whether a women was pregnant or breast feeding.

### Factors associated with epilepsy

Case control pairs were generated from individuals in the prevalence survey database by matching individuals from the same health area, of the same gender and birth year. In this case control sub-study, only cases were included who were old enough to be eligible for Ivermectin distribution the year before they became epileptic (at least 6 years old at epilepsy appearance), and who lived in health areas where Ivermectin was distributed under the CDTI program in the year before they became epileptic.

Univariate binomial logistic mixed regression models were constructed to identify whether there was an association between the epilepsy status and the individual Ivermectin treatment history. A random effect term was included for pair identity. For cases (persons with epilepsy), this was considered as Ivermectin treatment in the year immediately before epilepsy was identified, and the proportion of years where Ivermectin was received in the years before and after the reported onset of epilepsy. For controls (persons without epilepsy) the Ivermectin history was considered similarly, around the age at which their paired case became epileptic.

The association between epilepsy and potential risk factors, including the proportion of Ivermectin doses received on occasions where the individual was eligible and the presence of onchocerciasis-associated skin lesions, was assessed through the construction of a binomial logistic mixed regression model using the whole survey data base, without case control pairing. Age and gender were controlled for as categorical fixed effects, and the health area included as a random effect to control for clustering at this level.

### Spatial distribution

To identify presence of clusters of epilepsy cases, the Kulldorff's scan statistic [10] as implemented in SaTScan software (https://www.satscan.org/) was used. The spatial scan statistic tests for spatial randomness of cases over the identified region. It defines a set of potential cluster areas (spatial circles of varying size), each consisting of a collection of cases. The most “unusual” cluster is then identified using the likelihood ratio test statistic which is based on the alternative hypothesis that the risk of the disease is greater inside than outside the circle. The most likely cluster is the circle with the maximum likelihood. For the epilepsy dataset, cases were defined as individuals with epilepsy while controls are those without epilepsy. The Kuldorff’s scan statistic was performed for each of the three administrative regions separately: Ituri, Tshopo and Bas-Uele province.

### Software

All statistical analyses (aside from the scan statistic above) were implemented using the R statistical computing environment [11]. The generalized linear mixed model was implemented using the package “lme4” [12].

### Ethics statement

The study was approved by the Institutional Review Board of Ngaliema hospital in Kinshasa and the ethical review board of the University of Antwerp. Written informed consent was obtained from the head of the family and parents/guardians of children. For people who could not write, consent was obtained by finger printing.

## Results

**Fig 2:**
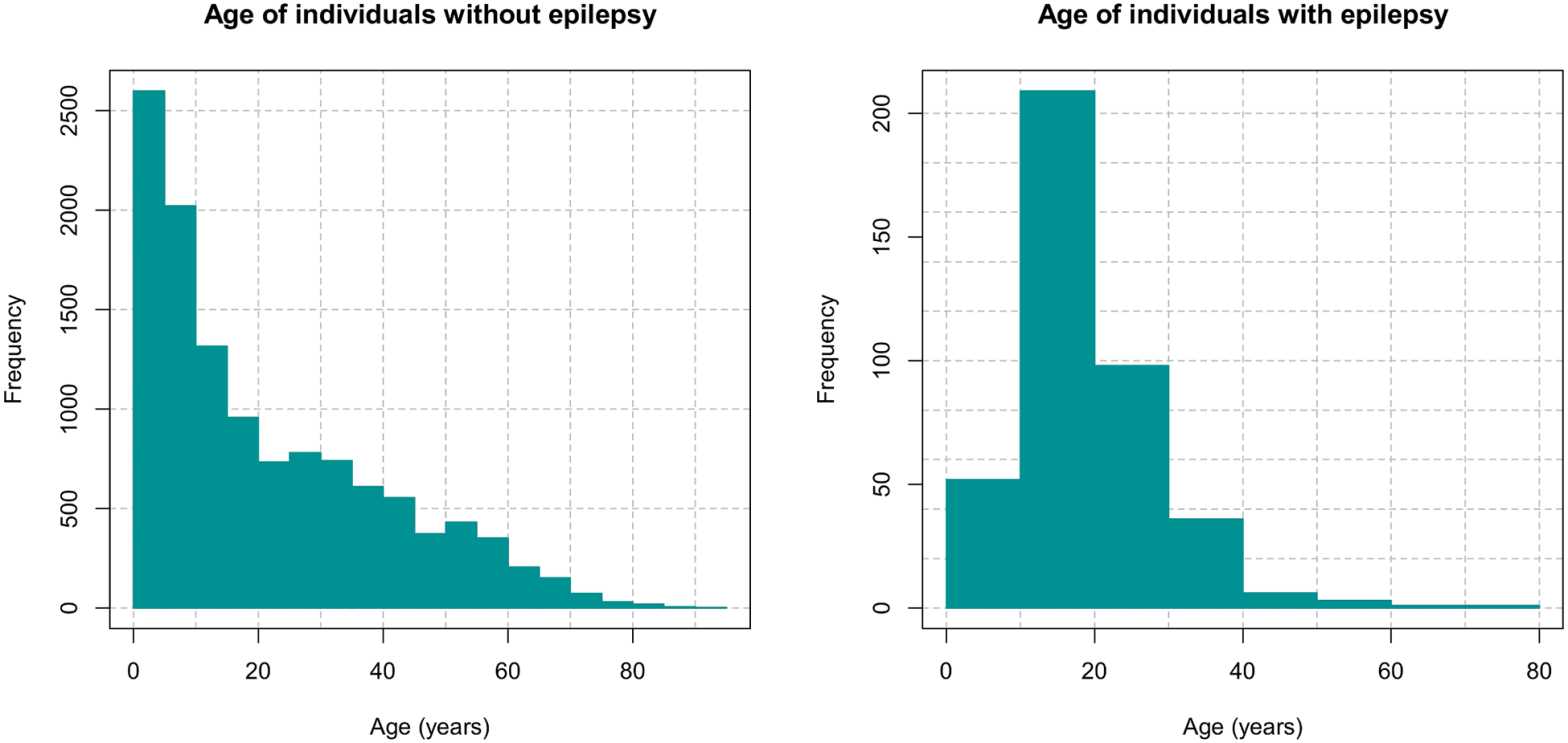
Histograms showing the distribution of ages in persons without (left) and with epilepsy (right).

Of the 12,408 people examined in the different health areas, 407 (3.3%) were found to have a history of epilepsy. Across the localities, the mean number of household members was 5.65 (s.d. = 3.35). Whilst the median ages of the persons with epilepsy (18 years, IQR = 13, 23.75) and without epilepsy (16 years, IQR = 6, 35) were relatively similar, the age distributions of the persons with and those without epilepsy were significantly different (Two Sample Kolmogorov-Smirnov test, D = 0.26, P<0.001) (Fig 2).

The median age of onset of epilepsy was 9 years; the modal age of onset was 12 years. The highest prevalence of epilepsy was observed in the 10–19 years age group (Table 1).

**Table 1:** Age-specific prevalence rates of epilepsy.

Age (years)	Population	Number of patients with epilepsy	Epilepsy prevalence (%)
**> 40**	2411	12	0.5%
**30–39**	1421	47	3.3%
**20–29**	1600	105	6.6%
**10–19**	2665	205	7.7%
**< 10**	4269	37	0.9%
**All ages**	12,366*	406*	3.3%

*42 individuals (of which one with epilepsy) had no age recorded and so are excluded.

**Fig 3:**
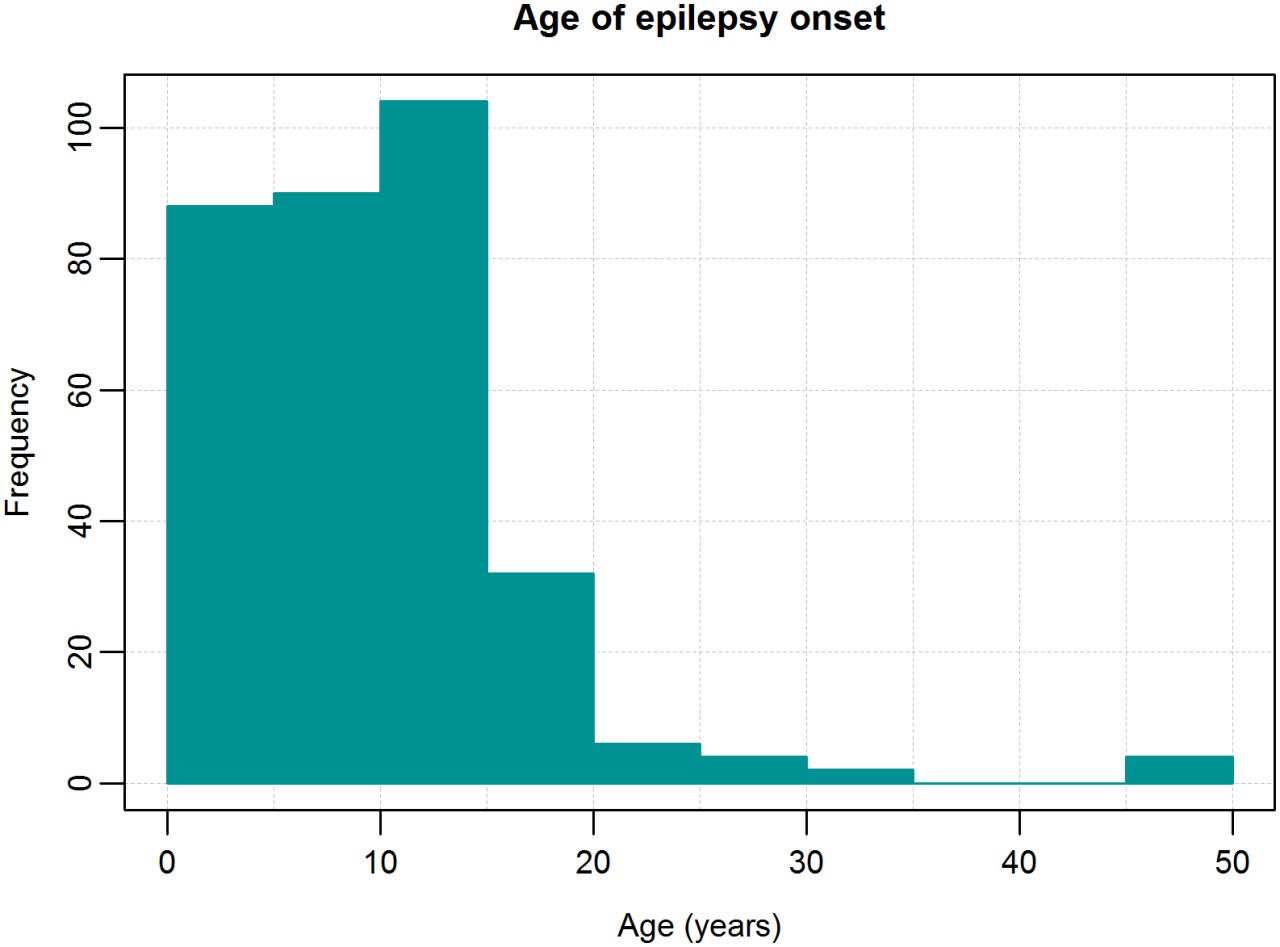
Histogram of age of epilepsy onset.

Epilepsy appeared most frequently in the 10–15 year age group (Fig 3).

In 41 families there were at least 2 persons with epilepsy (1.5% of all households), and in 9 families at least three (0.3% of all households). Amongst the persons with epilepsy, 30% (123) lived in households with another person with epilepsy. All persons with epilepsy had experienced seizures in the last 5 years.

A high prevalence of epilepsy was observed in all health areas in the 3 provinces: 6.8–8.5% in Bas-Uele, 0.8–7.4% in Tshopo and 3.6–6.2% in Ituri (Table 2).

**Table 2:** Prevalence rates of epilepsy, onchocerciasis (*O*.*v*.) endemicity, years of Ivermectin distribution and Ivermectin coverage in the surveyed health areas of Bas-Uele, Tshopo and Ituri Provinces.

Province	Health Zone	Health Area	Population Surveyed	Epilepsy prevalence	Epilepsy of recent onset^1^	*O*.*v*. nodules	*O*.*v*. skin lesions^6^	Years of Ivermectin distribution	Ivermectin coverage 2014^7^
Ituri	Logo	Draju	1062	66 (6.2%)	6 (1.0%)	17/52 (32%)^2^	5 (0.5%)	0	0%
	Rethy	Rassia	898	32 (3.6%)	3 (0.3%)	15/49 (30.6%)^2^	2 (0.2%)	3	649/739 (87.8%)
		Lokpa	843	31 (3.7%)	3 (0.4%)	5/50 (10%)^2^*	0.0%	3	524/718 (73.0%)
Tshopo	Yahuma	Mombongo	1260	26 (2.1%)	2 (0.2%)	57%^3^*	1 (0.1%)	10	312/945 (33.0%)
	Tshopo	Makutano Pumuzikia	203	15 (7.4%)	2 (1.0%)	94%^4^*	4 (2.0%)	9	26/148 (17.6%)
	Yaleko	Yatange	424	11 (2.6%)	1 (0.2%)	67%^4^*	17 (4.0%)	4	12/342 (3.5%)
	Wanierukula (PK30-PK70)	Salambongo	5657	147 (2.6%)	9 (0.2%)	8/43 (18.6%)^2^	104 (1.9%)	10	601/4182 (14.4%)
		Makana	1121	9 (0.8%)	0%	55%^4^*	5 (0.4%)	10	112/826 (13.6%)
Bas-Uele	Aketi	Wela	570	39 (6.8%)	6 (1.1%)	98%^5^*	55 (9.6%)	13	298/458 (65.1%)
		Makoko	367	31 (8.4%)	5 (1.4%)	98%^5^*	12 (3.3%)	13	217/278 (78.1%)

^1)^ Onset of seizures in the last 12 months

^2)^ REMO data in September 2015 by our research team

*data obtained from the PLNO

^3)^ RAPLOA, 2005.

^4)^ RAPLOA, 2003

^5)^ REMO, 1999 [13]

^6)^ Only Leopard type of skin lesions were considered as *O*.*v*. skin lesions.

^7)^ Number of individuals who reported to have taken Ivermectin in 2014/individual eligible to receive Ivermectin (%).

Ninety six cases and controls perfectly matched for village, age and gender were identified (Table 3).

**Table 3:** Health zone and area distribution of case control sample.

Health Area	Number of case control pairs from this health area
Rassia	4
Lokpa	5
Mombongo	4
Salambongo	35
Makana	4
Wela	21
Makoko	14
Yatange	2
Makutano Pumuzikia	7
Total number of pairs	96

Within the case control pairs, before the appearance of epilepsy, compared to the same life period in controls, persons with epilepsy had taken less frequently Ivermectin than controls (Table 4).

**Table 4:** Case control study: Ivermectin history according to epilepsy status, in health area, age and gender matched case and control study univariate analysis, with a random effect for pair identity.

Factor	Odds Ratio	95% C.I.	P Value
Ivermectin received the year before epilepsy appeared (equivalent year in the controls)	0.52	0.28, 0.98	0.04
Proportion of occasions, in years eligible, Ivermectin received before epilepsy appearance (or equivalent period in controls)	0.46	0.22, 0.95	0.04
Proportion of occasions, in years eligible, Ivermectin received after epilepsy appearance (or equivalent period in controls)	0.89	0.46, 1.70	0.71

After the appearance of epilepsy, there was no difference of Ivermectin intake between cases and controls.

Using the whole survey database, male gender, onchocerciasis skin lesions and being treated, at least once with Ivermectin, were associated with a significantly higher risk of being a person with epilepsy (Table 5).

**Table 5:** Multivariate regression analysis of individual risk factors for epilepsy.

Fixed effects		Population in sample	No. of patients with epilepsy	Epilepsy prevalence	OR	(95%CI)	P Value
Proportion of Ivermectin doses received					2.04	1.21, 3.42	<0.01
Onchocerciasis associated skin lesions (leopard skin)	Present	205	18	8.78%	4.57	2.34, 8.57	<0.001
	Absent	12,131	388	0.32%			
Interaction between proportion of Ivermectin doses received and presence of Onchocerciasis associated skin lesions (leopard skin)					0.08	0.003, 0.79	0.04
Age					0.99	0.98, 0.99	<0.001
Gender	Male	6041	221	3.66%	1.27	1.03, 1.56	0.01
	Female	6304	186	2.95%			
Health area random effect^1^					0.31		
Patient random effect^2^					0.54		

^1)^ Variance between random by-health area effect

^2)^ Variance between random patient effect

Among the patients without leopard skin, those who used Ivermectin were twice as likely (OR = 2.04) to have epilepsy as compared to those who did not use Ivermectin. However, among the patients with leopard skin, thus showing signs of onchocerciasis, those who used Ivermectin were 84% less likely (OR = 0.16) to have epilepsy than those who did not use Ivermectin.

**Fig 4:**
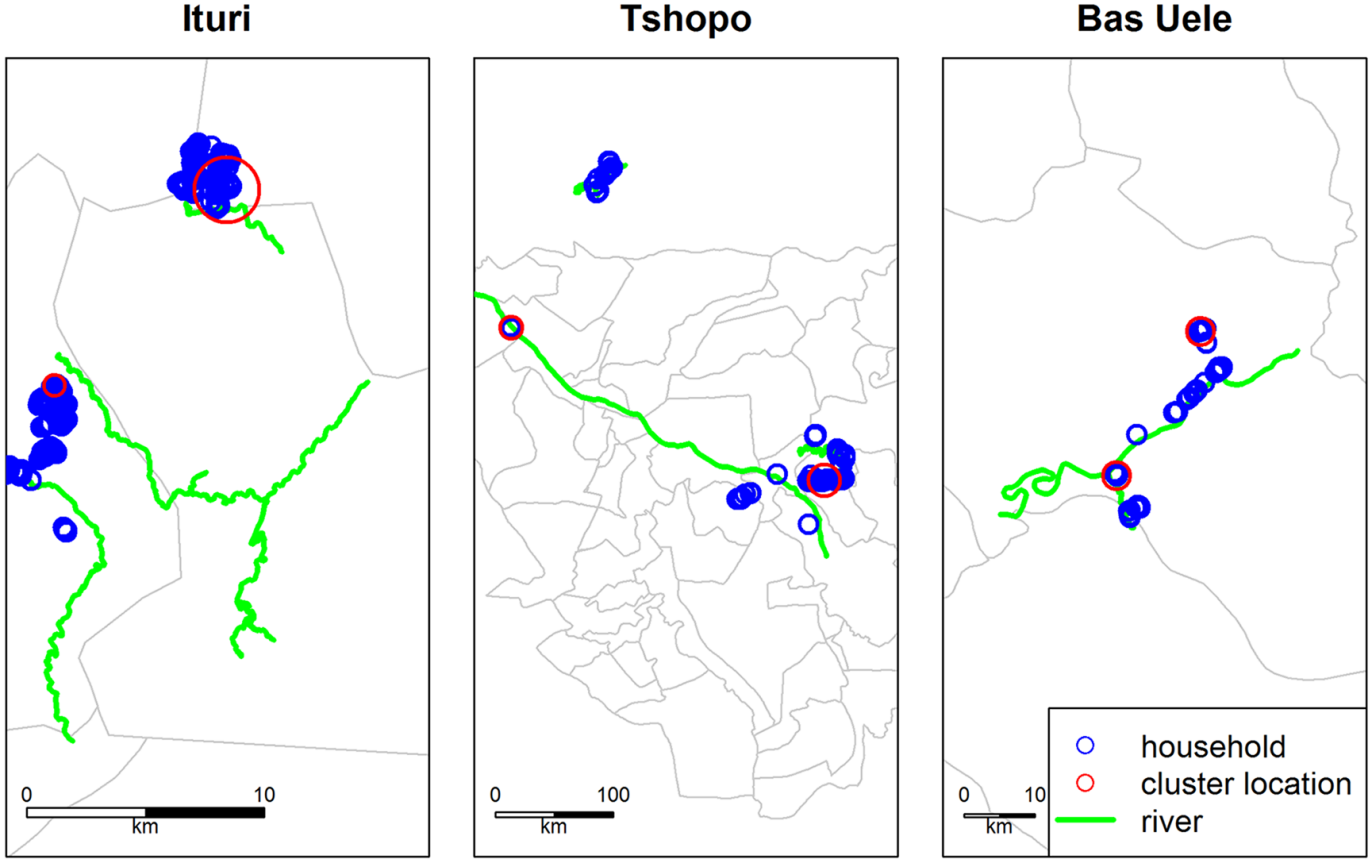
Plot of the 2 most likely clusters of epilepsy cases for each province, based on Kulldorff’s scan statistics. Each blue circle (seen here as overlapping due to close proximity) represents one household, regardless of the epilepsy status.

With regard to clustering of epilepsy cases, based on the Kulldorff scan statistics, 5 most likely clusters of epilepsy cases were detected in Ituri, 9 in Tshopo and 2 in Bas-Uele. However, most identified clusters had non-significant p-value (>0.1). In fact, of the 2 most likely clusters, one in Ituri and the other one in Tshopo (Fig 4), only one had p-value less than 0.05.

The cluster in Ituri had p-value of 0.0001 with a radius of 1.41 km and is located around the Draju sample site area with the Muda and Kuda rivers as nearest potential Simulid breeding sites; however the local vector remains to be identified. On the other hand, the cluster in Tshopo had p-value of 0.085 with a radius of 2.43 km and is located in between the Makana and Salambongo sample sites close to the Mobi and Onane rivers where members of the *Simulium neavei* complex were found on crabs. The most northern cluster in the Tshopo Province is located around the Tshopo rivers where *Simulium damnosum s*.*s*. were aggressively biting the villagers at the time of the study (data on blackflies exposure per province to be published elsewhere).

## Discussion

The observed prevalence of epilepsy in communities of onchocerciasis endemic regions in the DRC was comparable with those reported in other onchocerciasis endemic areas in Africa [14] but higher than the epilepsy prevalence (0.4–1%) reported in most studies in the rest of the world [15–19]. In a study of 586,607 residents in five Health and Demographic Surveillance System centres in sub-Saharan Africa, only 1,711 (0.29%) individuals were diagnosed as having active convulsive epilepsy [20]. However, we did observe large differences in the prevalence of epilepsy in different health areas, although the causes of these differences were not easy to explain. The localities involved in this study were spread across a large geographical (and ecological) range, which is likely to encompass different levels of risk of exposure to the blackfly vector. In the Ituri province, the prevalence of epilepsy was higher in the village where Ivermectin had never been distributed. In Aketi on the other hand, despite 13 years of CDTI and a relatively high coverage of Ivermectin, the prevalence and incidence of epilepsy and the prevalence of onchocerciasis skin lesions was high. Wela is a village situated only 100m from the Agu rapids on the Itimbiri river, a historically known *Simulium damnosum* breeding site. In 1999, in Wela, before the introduction of CDTI, 98% of the men examined presented with onchocerciasis nodules (REMO). A high *S*. *damnosum* density was observed in Aketi in 2016, but we did not investigate the infection rate of the flies. It would be important to investigate whether in the past in the Aketi health zone, the Iverrmectin therapeutic coverage has been much lower than the one reported in 2014. In this health zone, we plan qualitative research to obtain information about the adherence to the annual CDTI program and a case control study to investigate whether other parasitic infections such as cysticercosis could explain the high prevalence of epilepsy. Alternative hypotheses may be i) that in this health zone, the population of *O*.*v*. may have developed Ivermectin resistance, ii) a higher vectorial capacity of *S*. *damnosum sensu stricto* compared to other vectors (unidentified so far in the Ituri province and *S*. *damnosum*-complex in Tshopo province), iii) higher density of infective vector and hence an increased level of exposure to Simuliidae infective bites. The infestation rate of the *Simulium* spp. in the three foci would help answering some of these questions.

The peak incidence of epilepsy in patients in this study was around the age of 12. This aligns with other onchocerciasis endemic African regions, and can most likely be explained by the equally high incidence of *O*.*v*. infection in these age groups and the cumulative nature of the *O*.*v*. infestation [21]. This peak incidence is in contrast with the epilepsy situation in industrialized countries and in non-onchocerciasis endemic regions in Africa where most onset of epilepsy is observed in the very young (< 5 years old) and in the older population [21]. It is important to note that not only was there a high prevalence of epilepsy in the 10–19 age group, but also in the 20–29 age group. The latter is in contrast with the epilepsy prevalence reported in onchocerciasis hyperendemic regions where Ivermectin has not yet been introduced. For example, in a study from 1991 in Kyarusozi sub-county in western Uganda, 91% of the epilepsy cases were below the age of 19 [22]. This epilepsy age shift towards older age groups after several years of annual Ivermectin distribution is an argument that Ivermectin may reduce the incidence of epilepsy in children [23].

Around a third of the persons with epilepsy lived in a household with at least one other person with epilepsy. In a study in Europe, only 9.5% of persons with epilepsy had first- or second- degree relatives with seizures [24]. In Ituri, evidence of a significant clustered distribution of households with family members with epilepsy compared to those without epilepsy was observed. This aligns with observations from other onchocerciasis endemic areas [25] and most likely reflects shared exposure to infective bites of the local vector(s). The fact that only a relevant epilepsy cluster was observed in the Logo Health zone in Ituri may be because this was also the only zone, included in the survey, where in certain villages Ivermectin was never distributed. A (spatial) point process model in order to predict the risk of epilepsy due to the infective vector density could be of interest. However, this was not possible given the available data (sample locations were too irregular and clustered only in specific areas).

Similar to the results of a previous small case control study performed in Titule (Bas-Uele), the current case-control matched pairs analysis demonstrated that before the appearance of epilepsy, compared to the same life period in controls, persons with epilepsy had taken Ivermectin less frequently than controls. Considering the whole population, significant associations were identified between Ivermectin use, the presence of skin lesions and epilepsy status, even with fixed factor controlled for age and gender. Among patients with leopard skin, those who used Ivermectin were 84% less likely to have epilepsy than those who did not use Ivermectin. Leopard skin is a clinical sign that indicates that the person has been heavily exposed to *O*.*v*. and/or that the person did not take Ivermectin while infected with onchocerciasis. This finding may indicate that also when infected with onchocerciasis Ivermectin may reduce the risk for epilepsy. Among patients without leopard skin, those who used Ivermectin were twice likely to have epilepsy as compared to those who did not use Ivermectin. This could be explained by the fact that people use Ivermectin because of itching caused by onchocerciasis. People know that the itching disappears with the intake of Ivermectin. In a previous study, individuals reporting itching were more likely to be persons with epilepsy [6]. After the intake of Ivermectin, microfilariae disappear from the skin together with the itching and people stop scratching. Therefore, because of decreased scratch lesions and skin inflammation people taking Ivermectin are less likely to develop Leopard skin lesions but they are more likely to have been infected with the *O*.*v*. parasite in the past and therefore are more likely to have epilepsy. There are no arguments for suspecting that Ivermectin is able to cause seizures. Indeed, Ivermectin is not known to pass the blood brain barrier in humans. Only in certain dogs, collies and Australian shepherds because of MDR1 polymorphism, Ivermectin is able to cause seizures [26]. Ivermectin is rapidly absorbed. Therefore if Ivermectin could induce seizures, we would expect to see the onset of these seizures shortly after the administration of the Ivermectin. This was never reported despite the many millions of doses of Ivermectin that have been distributed for more than 20 years. Moreover, there is anecdotal evidence that in persons with onchocerciasis Ivermectin reduces the frequency of epilepsy [27]. In Aketi town, people were very eager to receive Ivermectin even more than once a year because the drug was known to reduce the itching and the community distributors treated themselves 3 to 4 times a year (Tepage F, personal communication).

Our study has several limitations. In most individuals, epilepsy was only reported and not observed. Only in the Makana health area (Tshopo) and Ituri (Logo and Rethy health areas), were individuals with epilepsy examined by a neurologist (JMK). A second neurologist, Dr D Mukendi recently visited the villages of Wela and Makoko and confirmed all the epilepsy cases that were identified during the 2015 survey. Laboratory investigations, to identify the cause of the epilepsy were not performed. In our definition of epilepsy, we did not specify a time limit for laps between seizures, and we mainly included convulsive epilepsy. Therefore, comparison of our results with published epilepsy prevalence data is difficult.

Additionally, the prevalence assessment was performed during short visits to the health zone with no accompanying qualitative studies. As discussed above, the exact nature of the relationship between Ivermectin use and epilepsy may be complex and as such it is likely to require knowing why people were taking or not taking Ivermectin. The different epilepsy surveys were performed with a similar methodology, however not always by the same research team. Certain data obtained during the surveys may suffer from recall bias. For example even age and age/year of onset of the epilepsy may be very imprecise. Questions could be raised about the reliability of the history of Ivermectin use, in certain individuals over a period of more than 10 years. Available REMO data are from 2003 and 2008 and are of little relevance after many years of CDTI. Indeed in the epilepsy case control studies we performed in Tshopo and Ituri a reduction in nodule carriers was observed.

In conclusion, the prevalence of epilepsy in villages in onchocerciasis endemic areas in the DRC was 2–10 times higher than in non-onchocerciasis endemic regions in Africa. Our study confirms previous findings that epilepsy is associated with *O*.*v*. infestation. Our data suggests that Ivermectin protects against epilepsy in an onchocerciasis endemic region. This finding suggests that in onchocerciasis endemic regions, the physiopathology of the epilepsy characterized by seizures starting between the ages of 3 to 18 years, is triggered by the *O*.*v*. parasite. Because microfilariae are not considered to invade the brain, the mechanism of this epilepsy could be through an auto-immune mechanism as was recently proposed for nodding syndrome [28,29]. However, a prospective population-based intervention study, ideally with Ivermectin twice a year, is needed to confirm that indeed Ivermectin protects against epilepsy in onchocerciasis endemic regions.

## Supporting information

S1 Questionnaire Questionnaire used for the prevalence survey. (DOCX)

S1 Checklist STROBE checklist. (DOCX)
