# Supplementary material for: High prevalence of epilepsy in onchocerciasis endemic regions in the Democratic Republic of the Congo
Source: PLoS Negl Trop Dis. 2017 Jul 14;11(7):e0005732. doi: 10.1371/journal.pntd.0005732 (PMC5529017; doi:10.1371/journal.pntd.0005732)
Supplement: S1 Questionnaire — (DOCX) [file pntd.0005732.s001.docx]

**EPILEPSIE DEPISTAGE.** Pour chaque personne du ménage pour laquelle de l’épilepsie a été RAPPORTEE ou OBSERVEE en page 1 du questionnaire ERPE

poser les 5 questions aux membres du ménage concerné. Si au moins 1 OUI, proposer de participer à l’étude et référer au neurologue.*IN : ne sait pas

**CODE UNIQUE du ménage : FA __ __ __ /WP__ __ __ / __ __ Division Province/__ __ __ Zone de santé / __ __ Aire de santé**

**Coordonnées GPS : LATITUDE (deg.dec) [S ou N] __ __ . __ __ __ __ __LONGITUDE [E ou O] __ __ . __ __ __ __ __ ALTITUDE _ __ __ __(en mètres)**

| **1.1. No. pers.**  **(si présente, cocher)** | **1.2. QUESTION 1**  Perte(s) de connaissance et / ou perte d'urine et / ou émission de bave ? | **1.3. QUESTION 2**  Absence(s) ou perte(s) de contact avec l'entourage de début brutal et de durée brève ? | **1.4. QUESTION 3**  Secousses ou mouvements  anormaux incontrôlables d'un  ou des membres (convulsions),  de début soudain et d’une  durée de quelques minutes ? | **1.5. QUESTION 4**  Apparition brutale et de durée  brève de sensations corporelles étranges, d’hallucinations ou  d’illusions visuelles, auditives  ou olfactives ? | **1.6. QUESTION 5**  A-t-on déjà dit au sujet qu’il  était épileptique ou qu’il avait  déjà fait **au moins 2**  **crises d’épilepsie ?** |
| --- | --- | --- | --- | --- | --- |
| **1** | **OUI NONIN*** | **OUI NONIN** | **OUI NONIN** | **OUI NONIN** | **OUI NONIN** |
| **2** | **OUI NONIN** | **OUI NONIN** | **OUI NONIN** | **OUI NONIN** | **OUI NONIN** |
| **3** | **OUI NONIN** | **OUI NONIN** | **OUI NONIN** | **OUI NONIN** | **OUI NONIN** |
| **4** | **OUI NONIN** | **OUI NONIN** | **OUI NONIN** | **OUI NONIN** | **OUI NONIN** |
| **5** | **OUI NONIN** | **OUI NONIN** | **OUI NONIN** | **OUI NONIN** | **OUI NONIN** |
| **6** | **OUI NONIN** | **OUI NONIN** | **OUI NONIN** | **OUI NONIN** | **OUI NONIN** |
| **7** | **OUI NONIN** | **OUI NONIN** | **OUI NONIN** | **OUI NONIN** | **OUI NONIN** |
| **8** | **OUI NONIN** | **OUI NONIN** | **OUI NONIN** | **OUI NONIN** | **OUI NONIN** |
| **9** | **OUI NONIN** | **OUI NONIN** | **OUI NONIN** | **OUI NONIN** | **OUI NONIN** |
| **10** | **OUI NONIN** | **OUI NONIN** | **OUI NONIN** | **OUI NONIN** | **OUI NONIN** |
| **11** | **OUI NONIN** | **OUI NONIN** | **OUI NONIN** | **OUI NONIN** | **OUI NONIN** |
| **12** | **OUI NONIN** | **OUI NONIN** | **OUI NONIN** | **OUI NONIN** | **OUI NONIN** |
| **13** | **OUI NONIN** | **OUI NONIN** | **OUI NONIN** | **OUI NONIN** | **OUI NONIN** |
| **14** | **OUI NONIN** | **OUI NONIN** | **OUI NONIN** | **OUI NONIN** | **OUI NONIN** |
| **15** | **OUI NONIN** | **OUI NONIN** | **OUI NONIN** | **OUI NONIN** | **OUI NONIN** |

**QUESTIONNAIRE PERSONNE SUSPECTE D’EPILEPSIE**

**DATE (JJ/MM/AAAA): ___ / ___/ ____NOM et PRENOM de l’enquêteur: ______________________________**

**Médecin  Neurologue  infirmier**

**LOCALITE : _________________________________________________________________________________**

**AIRE DE SANTE:_____________________________________________________________________________**

**NOM et PRENOM de l’INFIRMIER TITULAIRE :** ____________________________________________________

__________________________________________________________________________________________

| **Code UNIQUE du participant__ __ __ __ __ __ __ __ __ __ __ __ __ __ __ __ __ __ __ __**  **DONNEES SOCIO-DEMOGRAPHIQUES / PERSONNELLES** |
| --- |
| **1. Le répondant est-il le participant lui-même ?  OUI  NON**  **1.2. Si NON, quelle est la relation entre le participant et le répondant ? __________________________________________**  **2. Un traducteur a-t-il été nécessaire ?  OUI  NON**  **3. Depuis quand le participant vit-il dans le village? 1 2 3  Ne sait pas**  **(de passage = 1 ; moins de 1 an = 2 ; depuis plus de 1 ans = 3)**  **3.1. Si moins de 1 ans, nombre de mois : ____________mois**  **3.2. Si plus de 1 an, nombre d’années : ___________années**  **3.2. Si la personne vivait avant dans un autre village, dans quel village? ______________________________**  **3.4 Dans quelle AIRE de Santé ? ______________________________**  **3.6. Dans un autres pays ?  OUI  NON**  **Si dans un autre pays, spécifier : ________________________________________________________** |
| **4.1. Age : ______________ 4.2.Date de naissance, si connue : _____ / _____ / ______**  **4.3. Sexe :  M  F**  **4.4. Le participant est le quantième enfant né de la femme ? ______  NE SAIT PAS**  **4.5 Ethnie :     autre, si autre spécifier_________________________**  **4.6. Poids (kg) : _________ . ____kg**  **4.7. Taille (cm) : ___________cm** |
| **5.1. Niveau scolaire :**  **Aucun**  **Primaire  6i^eme^ 5e 4e 3e 2e 1e**  **Secondaire  6i^eme^ 5e 4e 3e 2e 1e^ème^**  **Supérieure**  **5.2. Activité principale de la famille:**  **aucune  agriculture  pêche  chasse  élevage  commerce**  **autre, spécifier : ________________________________________________________**  **5.3. Activité principale du participant:**  **aucune si enfant  agriculture  pêche  chasse  élevage  commerce**  **autre, spécifier : ________________________________________________________** |
| **6.1. Est-ce que le participant a déjà pris Ivermectine/Mectizan**  **OUI  NON  NE SAIT PAS**  **6.2. Si oui, combien de fois? _________ nombre de fois**  **6.3. Quand le participant a pris la dernière fois Ivermectine/Mectizan?**  **Année 20____ Mois_________**  **6.4. Si pas pris en 2016/17, pourquoi ?**  **pas distribué  absent durant le TIDC  refus  peur des effets secondaires**  **grossesse  allaitait un enfant de<7jours**  **parce qu’on m’a demandé de NE PAS le prendre  âge <5 ans au moment du TIDC**  **maladie grave**  **autre cause : spécifier _________________________________________________**  **6.5. Si pris en 2016/17, pourquoi ?**  **administration encouragée par les distributeurs de l’ivermectine**  **pour faire diminuer les démangeaisons**  **autre motivation, spécifier__________________________**  **6.6. Est-ce que l’Ivermectine/Mectizan a été utilisé pour une autre raison que recommandé par les distributeurs (par exemple pour utiliser sur la peau/cheveux)?  OUI  NON  NE SAIT PAS**  **Si oui, spécifier pourquoi ? ______________________________________________________________________** |
| **Code UNIQUE du participant__ __ __ __ __ __ __ __ __ __ __ __ __ __ __ __ __ __ __ __** |

| **ANAMNESE** |
| --- |
| **7.1. Questions concernant la naissance du participant enquêté, mère présente pour répondre aux questions ?**  **OUI  NON**  **7.2. Lieu de naissance du participant enquêté ?**  **Domicile  Centre de Santé  Hôpital  Autre  NE SAIT PAS**  **7.3. Accouchement :  normal ?  avec ventouse/forceps ?  par césarienne ?**  **A la naissance :**  **7. 4. Le participant enquêté est-il né prématuré ?  OUI  NON  NE SAIT PAS**  **7.5. Le participant enquêté a-t-il crié immédiatement ?  OUI  NON  NE SAIT PAS**  **7.6. Poids de naissance ? (en grammes) --------------grammes NE SAIT PAS**  **7.7. Est-ce qu’avant l’épilepsie, le participant a souffert de :**  **traumatisme crânien?  encéphalite/méningite?  neuro-paludisme ?  rougeole  maladie du sommeil ?  autre maladie grave  NE SAIT PAS?**  **7.8. si autre maladie, spécifier ____________________________________________________________** |
| **9.1. Convulsions fébriles dans l’enfance (0-10ans).  OUI  NON  NE SAIT PAS**  **9.2. Si OUI : à quel âge les convulsions fébriles ont-elles débuté ? _______________________** |
| **10.1 Perte de connaissance avec convulsions pas provoqué par la fièvre?  Jamais  1X  2X OU PLUS NE SAIT PAS** |
| **10.2. Perte(s) de connaissance avec perte(s) d’urine ?  OUI  NON  NE SAIT PAS** |
| **10.3. Perte(s) de connaissance avec émission de bave et/ou morsure de langue?  OUI  NON  NE SAIT PAS** |
| **10.4. Absence(s) ou perte(s) de contact avec l’entourage (participant ne répond pas, de début brutal et de durée brève) ?  OUI  NON  NE SAIT PAS** |
| **10.5. Episodes de hochement de tête avec perte de contact avec l’entourage (participant ne répond pas)?**  **OUI  NON  NE SAIT PAS** |
| **10.6. Secousses ou mouvements anormaux incontrôlables d'un ou des membres (convulsions, de début soudain et d’une durée de quelques minutes) ?  OUI  NON  NE SAIT PAS** |
| **10.7. Apparition brutale et de durée brève de sensations corporelles étranges, d’hallucinations ou d’illusions visuelles, auditives ou olfactives ?  OUI  NON  NE SAIT PAS** |
| **10.8. A-t-on déjà dit à la personne qu’elle était épileptique  ou qu’il avait déjà fait des crises d’épilepsie ?**  **OUI  NON  NE SAIT PAS** |

| **Code UNIQUE du participant__ __ __ __ __ __ __ __ __ __ __ __ __ __ __ __ __ __ __ __ __ __** |
| --- |
| **Si épileptique :**  **11.1. Age de début de l’épilepsie  : ______________**  **11.2. Année de début de l’épilepsie : ____________**  **11.3. Si depuis moins d’un ans, depuis combien de mois?___________NE SAIT PAS**  **11.4. Le participant a-t-il présenté une crise dans les 5 dernières années  OUI  NON  NE SAIT PAS**  **11.5. Le participant a-t-il présenté une crise la dernière année ?  OUI  NON  NE SAIT PAS**  **11.6. Le participant a-t-il présenté une crise le dernier mois ?  OUI  NON  NE SAIT PAS** |
| **Type d’épilepsie**  **12.1. Crises généralisées :  OUI  NON**  **12.2. Si oui,  tonico-cloniques  atoniques**  **12.3. Absences  OUI  NON**  **12.4. Crises partielles (focale) simple (crise sans de perte de connaissance) :  OUI  NON**  **12.5. Si oui  motrices  sensorielles**  **12.6. Crises partielles (focale) complexes (crise avec perte de connaissance)  OUI  NON**  **12.7. Crises secondairement généralisées  OUI  NON**  **12.8. Hochement de tête (avec période d’altération de l’état de conscience), non suivi immédiatement de convulsions  OUI  NON  NE SAIT PAS**  **12.9 Si hochement de tête ou autre forme d’épilepsie, quel facteur peut déclencher une crise ? (plusieurs réponses possibles)**  **pas de facteur spécifique la nuit le froid le repas les orages autre NE SAIT PAS**  **Si autre, spécifier :______________________________________** |
| **Avant le début de l’épilepsie**  Comparé aux enfants de même âge que lui, développement psycho-moteur **?**  **13.1. Capacité de marcher ?**  **tôt  même âge  plus tard  ne sais pas**  **13.2. Capacité de parler avec des phrases ?**  **tôt  même âge  plus tard  ne sait pas**  **13.3. Est-ce que l’enfant a grandi  plus vite  normalement  moins vite  ne sait pas**  **Après le début de l’épilepsie**  Comparé aux enfants de même âge que lui, développement psycho-moteur **?**  **13.4. Capacité de marcher ?**  **tôt même âge plus tard ne sais pas**  **13.5. Capacité de parler avec des phrases ?**  **tôt même âge plus tard ne sait pas**  **13.6. Est-ce que l’enfant a grandi  plus vite  normalement  moins vite  ne sait pas** |

| **14. Combien de crises d’épilepsie?**  **chaque jour (si plus que 30 par mois), combien de fois par jour :_________________fois/jour**  **chaque mois (si plus de 12 par an), combien de fois par mois : _________ fois/mois**  **chaque année (si moins de 12 par an) , combien de fois par an : _________ fois/an** |
| --- |
| **15.1. Est-ce que vous prenez des médicaments MODERNES contre l’épilepsie?**  **NON, jamais  OUI, de façon irrégulière   OUI, après chaque crise   OUI, continuellement**  **15.2. Si prise d’antiépileptiques modernes, lesquels ?**  **phénobarbital  carbamazépine  phenytoïne  valproate de sodium  Autre, spécifier: ____________________________________**  **15.3. Est-ce que vous prenez des médicaments TRADITIONNELS contre l’épilepsie?**  **NON, jamais  OUI, de façon irrégulière  OUI, continuellement** |
| **16.1 Est-ce qu’il y a d’autres personnes avec épilepsie dans la famille**  **OUI  NON  Ne sait pas**  **16.2. Si OUI, préciser :**   1. **père  mère** 2. **frère ou sœur : préciser le nombre : __________**   **16.3. Combien de membres de la famille souffrent d’épilepsie ? __________**  **16.4. Combien avec âge de < 20 ans __________**  **16.5. Le participant enquêté a-t-il un jumeau ou une jumelle identique ?**  **OUI NON NE SAIT PAS**  **16.6. Si oui, est-ce que le jumeau souffre d’épilepsie ? OUI NON NE SAIT PAS**  **16.7. Démangeaisons OUI NON**  **16.8. Insomnies à cause de démangeaisons OUI NON** |

| **EXAMEN PHYSIQUE** | | | | |
| --- | --- | --- | --- | --- |
| **17.1. ETAT GENERAL** | **Bonne santé  modérément altéré  mauvais** | | | |
| **17.2. VISION NORMALE** | **OUI** | | **NON** | |
| **17.3. ŒIL VITREUX** | **OUI** | | **NON** | |
| **17.4. AVEUGLE DES 2 YEUX** | **OUI** | | **NON** | |
| **17.4. LESIONS/CICATRICES DE BRULURES** | **OUI** | | **NON** | |
| **17.5. PROFONDEUR DES BRULURES** | **Score :** | | | |
| **17.6. RETRACTIONS DU AUX BRULURES** |  | |  | |
| **17.7. LESIONS TRAUMATIQUES** | **OUI** | | **NON** | |
| **17.8. PAPULES PRURIGINEUSES** | **OUI** | | **NON** | |
| **17.9. PEAU DE LEOPARD** | **OUI** | | **NON** | |
| **17.10. PEAU DE LEZARD** | **OUI** | | **NON** | |
| **17.11. DEMANGEAISONS** | **OUI** | | **NON** | |
| **17.12. PEAU NORMALE** | **OUI** | | **NON** | |
| **17.13. NODULES ONCHOCERQUIENS** | **OUI NON**  **17.11. Si OUI, nombre _______________________________** | | | |
| **18.1. CANDIDOSE BUCCALE** | **OUI NON** | | | |
| **18.2. SIGNES DE MORSURES DE LANGUE** | **OUI NON** | | | |
| **19. GANGLIONS CERVICAUX ANORMAUX (>1cm)** | | **OUI** | | **NON** |
| **20.1. ADOLESCENT (>16 ans) OU ADULTE QUI RESEMBLE A UN ENFANT** | | **OUI** | | **NON** |
| **20.2. Si OUI, ABSENCE DE SIGNES EXTERNES DE DEVELOPEMENT SEXUEL (caractères sexuels secondaires)** | | **OUI NON**  **21.3. Si OUI, spécifier: Filles: seins non développés**  **Filles et garçons: pas de poils sur le pubis** | | |
| **20.3. DEFORMATION CORPORELLE:  THORACIQUE  COLONNE VERTEBRALE  VISAGE**  **Si déformations, spécifier :_____________________________________________________________** | | | | |
| **21.1. CONSCIENCE  Normale  altérée** | | | | |
| **21.2. TROUBLE MENTAL ? NON Peu important  (dérange les activités mais encore actif)  Important (empêche d’exercer les activités habituelles)**  **21.3. Si trouble mental (important ou peu important): lequel? (plusieurs réponses possible)**  **difficultés de s’exprimer  désorientation hallucinations  exprime des idées délirantes**  **ne comprends pas ce qu’on demande  épisodes agressifs**  **oublie facilement  trouble de comportement peu d’intérêt ou plaisir dans presque toute activité**  **autre, spécifier :_________________________________________** | | | | |
| **22.1. Marche normale ?  OUI  base élargie  spastique  hémiplégique**  **22.2. Faiblesse musculaire ?  NON  généralisée  localisée**  **22.3. Paralysie?  OUI  NON**  **22.4. Si oui, spécifier :__________________________________________**  **22.5. Contractures ?  OUI  NON**  **22.6. EXAMEN NEUROLOGIQUE :  NORMAL  ANORMAL**  **___________________________________________________________________________________________________**  **23. Diagnostic  Epilepsie   Syndrome du hochement de tète**  **Hochement de tête et autre forme d’épilepsie**  **Autre diagnostic:   Convulsions fébriles récurrentes**  **Syncopes**  **Vertiges**  **Anémie sévère**  **Retard mental sans épilepsie   Problème psychiatrique sans épilepsie** | | | | |

| **24.1. AUTRES problème médical :  OUI  NON**  **24.2. Si OUI, spécifier : _______________________________________________________________________** |
| --- |

**Signature MD/personnel santé Date**
