# Supplementary material for: High prevalence of epilepsy in onchocerciasis endemic regions in the Democratic Republic of the Congo
Source: PLoS Negl Trop Dis. 2017 Jul 14;11(7):e0005732. doi: 10.1371/journal.pntd.0005732 (PMC5529017; doi:10.1371/journal.pntd.0005732)
Supplement: S1 Checklist — (DOCX) [file pntd.0005732.s002.docx]

STROBE Statement—checklist of items that should be included in reports of observational studies

|  | Item No. | Recommendation | Page  No. | Relevant text from manuscript |
| --- | --- | --- | --- | --- |
| **Title and abstract** | 1 | (*a*) Indicate the study’s design with a commonly used term in the title or the abstract |  | Prevalence study and nested case control study |
|  |  | (*b*) Provide in the abstract an informative and balanced summary of what was done and what was found | 2, 3 |  |
| Introduction | | | |  |
| Background/rationale | 2 | Explain the scientific background and rationale for the investigation being reported | 4 |  |
| Objectives | 3 | State specific objectives, including any prespecified hypotheses | 4 | High epilepsy prevalence in onchocerciasis endemic region and Ivermectin may be able to protect against epilepsy |
| Methods | | | |  |
| Study design | 4 | Present key elements of study design early in the paper | 5-7 | Prevalence study and nested case control study |
| Setting | 5 | Describe the setting, locations, and relevant dates, including periods of recruitment, exposure, follow-up, and data collection | 5-7 |  |
| Participants | 6 | (*a*) *Cohort study*—Give the eligibility criteria, and the sources and methods of selection of participants. Describe methods of follow-up  *Case-control study*—Give the eligibility criteria, and the sources and methods of case ascertainment and control selection. Give the rationale for the choice of cases and controls  *Cross-sectional study*—Give the eligibility criteria, and the sources and methods of selection of participants | 6-7 | Cases: persons with epilepsy, Controls: persons without epilepsy.  Needed to be eligible for ivermectin intake |
|  |  | (*b*) *Cohort study*—For matched studies, give matching criteria and number of exposed and unexposed  *Case-control study*—For matched studies, give matching criteria and the number of controls per case |  | Same health area, of the same gender and birth year. |
| Variables | 7 | Clearly define all outcomes, exposures, predictors, potential confounders, and effect modifiers. Give diagnostic criteria, if applicable | 6-9 | Prevalence epilepsy, GIS location, ivermectin use  ILAE definition of epilepsy |
| Data sources/ measurement | 8* | For each variable of interest, give sources of data and details of methods of assessment (measurement). Describe comparability of assessment methods if there is more than one group | 6-9 | Interview of individuals, clinical exams of persons with epilepsy |
| Bias | 9 | Describe any efforts to address potential sources of bias | 7-8 | There was certainly a recall bias about the use of ivermectin. However we expect a similar bias occurred in cases and controls |
| Study size | 10 | Explain how the study size was arrived at | 6-7 | The total number of persons interviewed and examined was large, however in certain villages the number of people included in the survey was small because the village was small or because of feasibility (limited time to perform the survey). The 96 cases and controls were all the cases and controls in the data set for which a perfect matching was possible. |

Continued on next page

| Quantitative variables | 11 | Explain how quantitative variables were handled in the analyses. If applicable, describe which groupings were chosen and why |  |  |
| --- | --- | --- | --- | --- |
| Statistical methods | 12 | (*a*) Describe all statistical methods, including those used to control for confounding | 7-8 |  |
|  |  | (*b*) Describe any methods used to examine subgroups and interactions | 7-8 |  |
|  |  | (*c*) Explain how missing data were addressed | 7-8 |  |
|  |  | (*d*) *Cohort study*—If applicable, explain how loss to follow-up was addressed  *Case-control study*—If applicable, explain how matching of cases and controls was addressed  *Cross-sectional study*—If applicable, describe analytical methods taking account of sampling strategy |  |  |
|  |  | (*e*) Describe any sensitivity analyses |  |  |
| Results | | | | |
| Participants | 13* | (a) Report numbers of individuals at each stage of study—eg numbers potentially eligible, examined for eligibility, confirmed eligible, included in the study, completing follow-up, and analysed | 8-11 |  |
|  |  | (b) Give reasons for non-participation at each stage |  | Some people not present in the household |
|  |  | (c) Consider use of a flow diagram |  |  |
| Descriptive data | 14* | (a) Give characteristics of study participants (eg demographic, clinical, social) and information on exposures and potential confounders | 8-11 | Survey: randomly selected people in the village  Case control: Ivermectin eligible individuals |
|  |  | (b) Indicate number of participants with missing data for each variable of interest | 9 | Of the 12,408 people examined 42 individuals (of which one with epilepsy) had no age recorded and so are excluded. |
|  |  | (c) *Cohort study*—Summarise follow-up time (eg, average and total amount) |  |  |
| Outcome data | 15* | *Cohort study*—Report numbers of outcome events or summary measures over time |  |  |
|  |  | *Case-control study—*Report numbers in each exposure category, or summary measures of exposure | 11 | 96 cases and controls |
|  |  | *Cross-sectional study—*Report numbers of outcome events or summary measures | 10-12 |  |
| Main results | 16 | (*a*) Give unadjusted estimates and, if applicable, confounder-adjusted estimates and their precision (eg, 95% confidence interval). Make clear which confounders were adjusted for and why they were included | 11-12 | Village, age and gender  These variables influence the risk of onchocerciasis |
|  |  | (*b*) Report category boundaries when continuous variables were categorized |  |  |
|  |  | (*c*) If relevant, consider translating estimates of relative risk into absolute risk for a meaningful time period |  |  |

Continued on next page

| Other analyses | 17 | Report other analyses done—eg analyses of subgroups and interactions, and sensitivity analyses | 13 | Spatial analysis |
| --- | --- | --- | --- | --- |
| Discussion | | | | |
| Key results | 18 | Summarise key results with reference to study objectives | 13-15 |  |
| Limitations | 19 | Discuss limitations of the study, taking into account sources of potential bias or imprecision. Discuss both direction and magnitude of any potential bias | 16 |  |
| Interpretation | 20 | Give a cautious overall interpretation of results considering objectives, limitations, multiplicity of analyses, results from similar studies, and other relevant evidence | 16-17 |  |
| Generalisability | 21 | Discuss the generalisability (external validity) of the study results | 16-17 |  |
| Other information | |  | | |
| Funding | 22 | Give the source of funding and the role of the funders for the present study and, if applicable, for the original study on which the present article is based |  |  |
